# What makes a microsaccade? A review of 70 years of research prompts a new detection method.

**DOI:** 10.16910/jemr.12.6.13

**Published:** 2020-03-17

**Authors:** Anna-Katharina Hauperich, Laura K. Young, Hannah E. Smithson

**Affiliations:** University of Oxford, UK

**Keywords:** fixational eye movements, microsaccades, automated detection, eye tracking

## Abstract

A new method for detecting microsaccades in eye-movement data is presented, following a review of reported microsaccade properties between the 1940s and today. The review focuses on the parameter ranges within which certain physical markers of microsaccades are thought to occur, as well as any features of microsaccades that have been stably reported over time. One feature of microsaccades, their binocularity, drives the new microsaccade detection method. The binocular correlation method for microsaccade detection is validated on two datasets of binocular eye-movements recorded using video-based systems: one collected as part of this study, and one from Nyström et al, 2017. Comparisons between detection methods are made using precision-recall statistics. This confirms that the binocular correlation method performs well when compared to manual coders and performs favourably compared to the commonly used Engbert & Kliegl (2003) method with subsequent modifications (Engbert & Mergenthaler, 2006). The binocular correlation microsaccade detection method is easy to implement and MATLAB code is made available to download.

## Introduction

**Figure 1.**
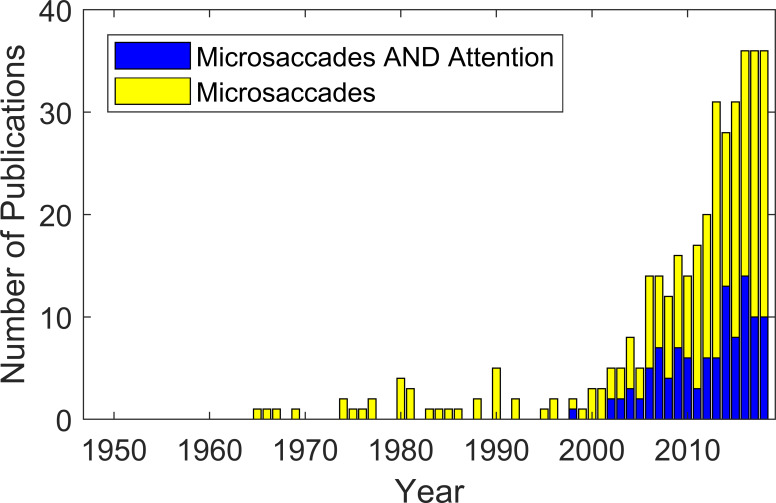
Numbers of papers that contain either the search term ‘microsaccades’ (yellow) or ‘microsaccades AND attention’ (blue) in abstract, title or keyword searches in Scopus, from 1948 to 2018. It should be noted that papers prior to 1996 may not be searchable via Scopus. Indeed a substantial number of papers prior to that date are included in our review, but not in the database search. However, the general trend since 1996 is evident.

Microsaccades have been the object of systematic study for over 70 years and still their correct identification remains difficult. Over time the focus of research on microsaccades has shifted, from trying to learn about their properties and purpose, to using them as a measure of cognitive processes such as covert attention (1, 2, 3). With an increase in the number of published papers on microsaccades (see Fig. 1) the central problem of how to detect microsaccades in a stream of eye-position samples is more important than ever.

Accurate identification of microsaccades from eye-movement traces rests on the specification and detection of characteristic signatures that are present in the traces. The broad characterisation of microsaccades as rapid, jerky movements points to some candidate features of the eye-movement trace – such as duration, peak velocity and peak acceleration – that may be candidate features for use in microsaccade detection methods. In addition, knowledge of the neural control of microsaccades might further inform the selection of defining features in the eye-movement trace.

To provide a formal basis for improving microsaccade detection, research articles from the past 70 years have been reviewed to identify which features of microsaccades have retained stable parameter estimates over this reporting period. We argue that this qualifies those parameters as candidates for use in microsaccade detection methods. Additionally, to encourage further research and discussion, we also describe microsaccade features that have not been consistently reported in the literature, or for which there is wide variability in parameter estimates.

One feature of microsaccades that we propose could provide a robust measure for their classification is binocularity. This is motivated specifically by two recent articles that have re-examined the evidence for the existence of monocular microsaccades (4, 5) and concluded that they are either artifacts of detection, or are exceedingly rare.

The first half of the paper summarises the main findings from the systematic literature review. The second half of the paper presents and formally evaluates a binocular correlation method for detecting microsaccades that is based on the simultaneous nature of microsaccades in both eyes. An advantage of using this binocular correlation method is its ease of implementation, and its roots in our understanding of the defining characteristics of microsaccades.

The performance of the binocular correlation (hereafter BC) method is compared to the Engbert and Kliegl (2003, hereafter EK) method with subsequent modifications by Engbert and Mergenthaler (2006), which is an automated detection method that is widely used and accepted. The comparative strengths and weaknesses of the two methods are discussed.

## Literature Review

### Methods

We were interested in learning about the physical parameter space spanned by microsaccades, and compiled a list of central criteria, which we extracted from the literature (see Table 1). Rolfs’ (2009) review of microsaccades was used to identify a set of papers to review between 1948 and 2009. Of those papers we compiled a list of papers with experimental data from human participants that include at least one piece of information from our list of central criteria. This resulted in a list of 53 articles.

**Table 1:** Selected data from the literature review on microsaccades. For the full table please refer to the supplementary methods.

**Paper**	**Analysis Method**	**Eye Tracker**	**Number of Participants**	**Min Distance [']**	**Mean Distance [']**	**Max Distance [']**	**Min Speed [°/s]**	**Mean Speed[°/s]**	**Max Speed[°/s]**	**Mean Acceleration [°/s²]**	**Max Acceleration [°/s²]**	**Min Duration [ms]**	**Mean Duration [ms]**	**Max Duration [ms]**	**Min Frequency [1/s]**	**Mean Frequency [1/s]**	**Max Frequency [1/s]**
Cherici, Kuang, Poletti, & Rucci, 2012	TH	DPI	14	17†	40	67†									0.16†	1.07	2.14†
Dimigen et al., 2009	EK	VB	12	**1.8**	13.8	**60**	**3**	/	**65**	/	/	/	/	/	**0.2**	1.46834	**6.5**
Ditchburn & Ginsborg, 1953	M	CLM	2	5	6.1/5.8	15	/	10	/	/	1000	/	/	/	0.2	.9-1.4	33.33
Martinez-Conde, Macknik, Troncoso, & Dyar, 2006	MC	VB	8	**1**	**14.4**	**120**	**3**	/	**40**	/	/	/	/	/	**3.2**	ng	**4.5**
McCamy et al., 2012	EK	VB	6	**1.8**	/	**60**	**5**	/	**140**	/	/	/	20	/	**0.5**	1.07	**2.6**
Møller et al., 2002	TH	VB	10	**6**	13-66	**150**	/	19-40	28-98	2321-6439	/	/	/	/	0.23†	0.61	0.93†
Otero-Millan et al., 2008	EK	VB	8	**2.4**	24.6	**60**	**10**	/	42	/	/	**4**	13	**34**	/	0.8	/
Otero-Millan et al., 2012	EK	VB	8	**3**	30	**153**	**15**	28 (peak)	**200**	/	/	4.3†	8	12†	0.48†	0.8	1.3†
Schulz, 1984	/	CLM	6	3	/	50	8.3	/	35	/	/	16	/	25	1	/	3
Siegenthaler et al., 2014	EK	VB	11	**6**	27	**120**	**10**	/	**170**	/	/	/	/	/	/	1.35	/
Troncoso, Macknik, & Martinez-Conde, 2008	EK	VB	6	**1.8**	24.6	180	/	41 (peak)	**100**	/	/	**2**	13	**101**	**0.2**	0.7	**2**
Valsecchi et al., 2007	EK	VB	17	**2**	/	90	**3**	/	**290**	/	/	/	/	/	0.733	1.544-1.797	**3.1**
Winterson & Collewijn, 1976	/	SC	7	**1**	7	**60**	/	/	/	/	/	/	/	/	0.079	1.25	1.78
Zuber & Stark, 1965	/	L	1	**2**	/	**13**	**3**	/	**14**	/	/	/	/	/	/	/	/
Hauperich, Young & Smithson	BCρ	VB	5	0.1	34	352	6.3	15.2	43.3	2800	5700	2	30	239	0.8	1.1	1.4
Hauperich, Young & Smithson	EK	VB	5	0.2	25	1218	10.2	28.8	62.5	3600	9200	3	25	92	1.4	2.4	3.4
Hauperich, Young & Smithson	M	VB	5	2.6	28	633	2.6	22.9	441	3300	6800	11	34	165	0.55	0.85	1.12

Note: The articles selected to appear in this table were chosen for the completeness with which they report the parameters we list in the columns of the table, as well as to represent a spread of papers through time. Data in bold represent values read off graphs, daggers indicate that the value given represents a median. In the column ‘Analysis method’ EK refers to variants of the Engbert and Kliegl (2003) method; MC refers to the criteria used by Martinez-Conde et al (2000); M indicates manual coding; TH refers to other threshold methods and BC indicates the BCρ method. In the column ‘Eye Tracker’ DPI refers to Dual Purkinje Image systems; VB indicates video-based eye tracking; CLM refers to contact lens mirror systems; SC refers to scleral coil systems and L refers to limbus trackers.

For papers published between 2009 and 2018 the Scopus database was searched using the term ‘microsaccades’ for ‘Article title, Abstract, Keywords’ (date of retrieval: 8^th^ Oct. 2018). The results were narrowed by using the Scopus inbuilt tool to select only documents listed under ‘Articles’ written in English. This left us with a list of 204 documents. Any papers without experimental data that had been included, mainly those focused on modelling, as well as any papers without human participants were excluded manually. The remaining articles were studied in order of most cited to least cited, stopping after having found 20 articles containing details featured in our central criteria. We limited this sample to 20 articles as we did not want to over-represent the most recent research in our analyses.

**Figure 2.**
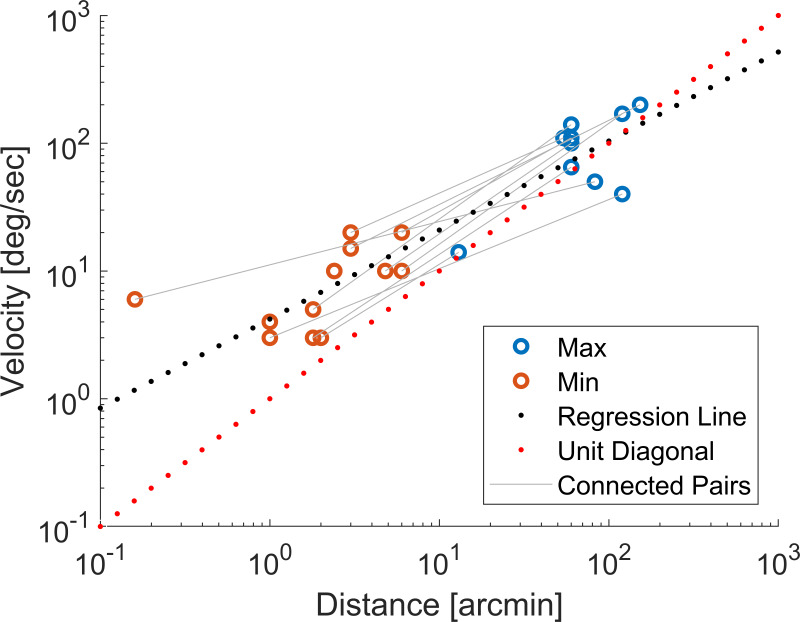
Minimum and maximum values of peak velocity and distance as extracted from the reviewed literature. The blue dots indicate the reported maximum velocities and the red dots the reported minimum velocities. Maximum and minimum points from the same publication are linked by a grey line. The red dotted line shows the unit diagonal while the black dotted line shows a regression line fitted to the data. To ensure that distance and velocity samples correspond to the same saccade only values read from graphs are included. The grey lines connecting one orange to one blue point represent samples reported in the same publication.

**Figure 3.**
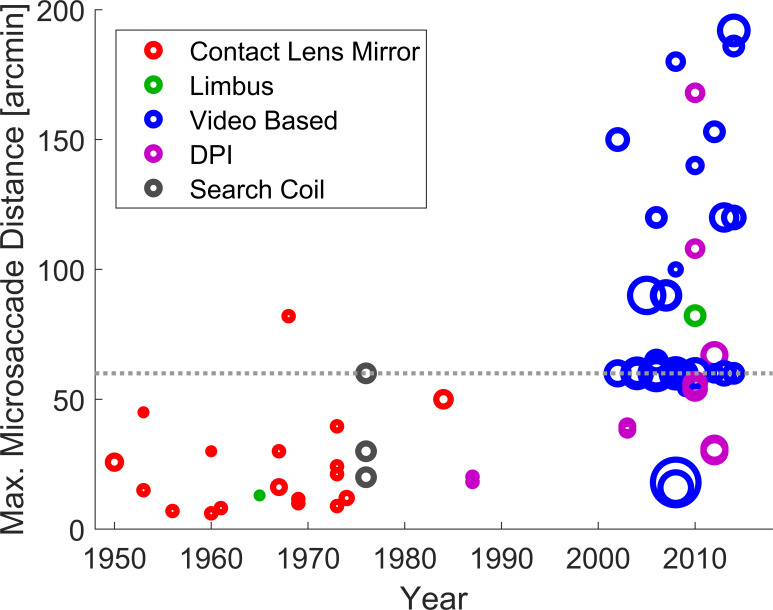
Maximum microsaccade size by year of publication. The area of each circle is scaled in relation to the number of participants. The circle colour represents the type of eye tracker. The dashed grey line indicates a 1° microsaccade.

In total 73 articles contributed to the data presented in Figures 2 and 3. Data from a subset of articles are presented in Table 1, and a full version featuring all 73 articles is available as supplementary information online.

### Results

Microsaccade research has experienced one major change in its history: the shift from historical eye trackers, which relied heavily on complex optical setups, to modern video-based eye trackers that depend on the underlying image-processing software for tracking. The greater ease of recording large amounts of eye tracking data from many different participants using video-based eye trackers brought with it changes in the types of questions investigated, and the way the collected data are processed.

Initially, eye trackers predominantly used variants of the contact lens mirror method (e.g. 7, 8, 9), which requires custom-made tight-fitting hard contact lenses to be worn by participants. These provided high temporal and spatial resolution eye tracking when the angle of reflection of a beam of light from the mirror on the contact lenses is recorded. This technique limited participant comfort (10) and “the cost of providing a large number of subjects with contact lenses is prohibitive” (11). Thus, only small numbers of participants could be tested for each experiment. Over time, other techniques that allowed for the testing of more participants and greater comfort during tracking became popular. These include (i) limbus trackers, which track the change in intensity of light reflected from the between the iris-sclera boundary (e.g. 12, 13), but often have low spatial resolution; (ii) search coils, which record the current induced by an external magnetic field in a coil of wire worn on the eye (e.g. 14), but still require large, uncomfortable lenses to be worn; and (iii) Dual Purkinje Image (DPI) trackers, which compare the first and fourth Purkinje reflections to calculate eye position (e.g. 15, 16). DPI trackers are the current gold standard for non-invasive high-resolution eye tracking, but require specialist knowledge to use and maintain.

Since the late 1990s video-based eye trackers, which analyse video data of the eye to extract gaze position, have become available. They are now widely used (e.g. 17, 18) due to their comparatively easy setup and maintenance, allowing for data collection on many different participants with relative ease. Whilst more convenient than older eye tracking methods, they can be limited in spatial and temporal resolution. Eye tracking remains a compromise between tracking resolution and accuracy, and participant comfort.

Video-based eye trackers have made eye tracking more readily accessible outside the specialist oculomotor community. Now microsaccades are used as a tool by cognitive scientists as a measure of cognitive processes, such as attention (see Fig. 1). 

Automated microsaccade detection became essential in dealing with the large amounts of data that could be collected, as manually coding each microsaccade became too time intensive. Several automated detection methods have been put forward, with the most widely used being the EK adaptive velocity threshold method. Others include methods combining velocity and linearity thresholds (19) and unsupervised clustering of velocity and acceleration samples (20). More recent methods also make use of Bayesian statistics (21) or neural networks (22). An ideal microsaccade detection method would correctly identify all microsaccade periods, be easy and fast to implement and be driven by our theoretical understanding of what constitutes a microsaccade.

Over the last 70 years some features of microsaccades have been consistently reported in different setups and tasks. It has been established that microsaccades follow the same main sequence relationship between their peak velocity and displacement as larger saccades (13). However, there appears to be some inconsistency in whether this relationship is plotted on linear or logarithmic coordinates. A linear relationship on logarithmic coordinates implies a power law relationship between the underlying linear variables (peak velocity and distance), with the gradient of the line representing the exponent. Therefore, a linear main sequence on logarithmic coordinates only represents a linear relationship between peak velocity and distance when the gradient of the line is equal to one.

Early papers consistently plot the relationship on log-log coordinates for both microsaccades (13) and large saccades (23). It is only in some more recent publications that it is sometimes plotted on linear coordinates (24, 25), with some even plotting it in both (4, 26). Over the distance and velocity ranges that can be measured with current eye trackers, the main sequence does appear to be linear for most reported data. However, plotting the data consistently on a log-log scale may be more informative, particularly as tracking methods and algorithms develop. This is because not only is it easier to spot systematic deviations from linearity, but it is also easier to evaluate the smallest and slowest microsaccades, which are most prone to errors, as they will be more spread out on a logarithmic scale. The main sequences for all data points extracted from the reviewed papers appears to be reasonably consistent across participants and setups (see Fig. 2), supporting the notion that the main sequence is a stable feature of microsaccades.

Another consistent finding in the literature is the variability of microsaccade rate with task instructions. First reported by Steinman and colleagues, it is a well-replicated effect that microsaccade rate declines with instructions to ‘hold’ the eye in place (27), with increased mental load (28) or in high acuity tasks (14, 29) (though note recent work by 30, 31), whilst under ‘fixate’ instructions, no instructions or a low mental load microsaccades occur more frequently. Microsaccade frequency is also influenced by target onsets, which lead to a reduction in microsaccade frequency (1, 32, 33). Modifying microsaccade rate using these methods is unlikely to provide improvements in microsaccade detection methods. However, using data that should display a change in microsaccade frequency could be a useful tool for evaluating microsaccade detection methods: if a detection method fails to reveal the expected patterns in the data, it is unlikely to be a robust method.

Microsaccades are also frequently described as ballistic in nature. This term has led to some confusion about the expected trajectories of microsaccades. When an object is moving ballistically it is subject to an initial force that sets it in motion until this motion is stopped by forces acting against it, such as friction or gravity. Similarly, microsaccades are hypothesised to be ballistic; they are set in motion by the eye muscles (34) following an initial cortical signal, after which their trajectory cannot be altered, as shown by double-step tasks for large saccades (35). However, this does not necessarily mean that their actual trajectories are linear in space. Ballistic motion can still result in complex curved paths (e.g. a ball being thrown follows a parabolic trajectory). Another source of uncertainty is the eye tracker, which may also have non-linearities distorting recorded eye positions and potentially leading to distorted path recordings. Despite this many researchers have described microsaccade paths as linear (1, 36, 37) or imposed linearity criteria on their microsaccade detection (38). As shown by examples of real recorded microsaccade paths, there are many different recorded trajectories ranging from linear paths (39) to paths with “significant curvature” (5). Whilst microsaccade paths are smoother than the zigzagging of tremor superimposed on drift, it would be wrong to assume that microsaccade trajectories are strictly linear. Given the complex, potentially curved nature of microsaccade trajectories it is difficult to use the ballistic property of microsaccade mechanics in detection methods, since an accurate model of the trajectory can only be achieved if we know all of the forces acting on the eye.

The emerging consensus is that binocularity (resulting in simultaneous, though not necessarily conjugate, or directionally correlated motions in the two eyes) is another defining feature of microsaccades. Conversely, drift and tremor are thought not to contain any significant temporally co-ordinated components in humans (40) (though this is still controversial, for a review see 6). The earliest recordings of binocular fixation data already showed a clear correlation between microsaccade occurrences in each eye (40). Significant correlations between the direction and magnitude of microsaccades in the two eyes have also been reported (41), as the majority of microsaccades are made in the same direction, with a subset being convergent, or even rarer, divergent (42). More recently monocular microsaccades have been investigated (43). However, it is very likely that any reported monocular microsaccades are artefacts of the recording and automated detection methods (5). In particular, it would appear that some tracker and algorithm combinations result in more frequent classifications of microsaccades as monocular, for example during head-free viewing (4). Imperfect head-stabilisation always comes with the risk of unwanted artefacts in the eye position trace. Perfect stabilisation is hard to achieve, but stabilisation spans a spectrum of security from the use of a bite bar, to using a head and chin rest to head-free viewing. As uncorrected head motion can lead to spikes in velocity in both eyes, neither velocity- nor binocularity-dependent methods are immune to such artefacts. Yet, as binocularity appears a fundamental and defining feature of microsaccades, it is a promising avenue for an automated detection method, whenever binocular eye tracking is available. 

Other reported aspects of microsaccades have changed considerably over time. The reported size of microsaccades has increased (44) from some as small as 10’ (14) and 15’ (29), to some as big as 60-120’ (2, 18, 30, 45, 46, 47, 48, 49). Fig. 3 illustrates this change in the largest reported microsaccade sizes, although it should be noted that the large spread of maximum distances reported since 2000 is in part caused by a change in the convention by which microsaccades are separated from larger saccades. Some studies set a hard upper limit, which results in a cluster of papers with a maximum size of 1°, whilst others reported all saccades (micro- and large) that happened during a fixation task. In cases where both an upper limit and all saccades during fixation were reported, we decided to plot the largest value, so as not to be influenced by the adequacy of a cut-off point.

It is suggested that there is no hard boundary between fixational saccades and large saccades, as they appear to be supported by the same neural systems (50) and display many similar properties (13, 32, 51, 52). Therefore, any saccades made during fixation should be treated as microsaccades. However, in some circumstances it may be questioned if some of the larger saccades in datasets could be target-directed. For example, the edge of a monitor or other features in the visual scene, not controlled within the experiment, might attract the participant’s gaze and therefore an exclusion threshold might be warranted. Even with a fixation task, the size of the fixation target may need to be considered as saccades might be made to different parts of the visual stimulus (38). There are several possible ways to define the maximum size of a microsaccade based on experimental data: Bridgeman and Palca decided on ‘the maximum size of stimulus displacement which does not normally induce a subject to perform a saccadic retargeting of the eye’. Other candidates could be the maximum size of saccades participants fail to self-report (53) or a direct reference to the size of relevant retinal anatomy, e.g. the foveola.

The theory that microsaccades are predominantly correcting for errors in eye position has enjoyed popularity for the last 70 years (for a detailed discussion see 54). Steinman, Haddad, Skavenski and Wyman (1973), however, showed that instead of microsaccades, a component of the motion trace named ‘slow control’, a type of drift, is mainly responsible for keeping the eyes on target with others reporting similar results (56, 57). Although some microsaccades, perhaps most, return the eyes closer to target position (58), this is not true for a large proportion of them (59), and the return to target is marked by significant overshoot (60). Despite slow control being an interesting target for research on the neural control of eye position, little has been reported on slow control since the advent of video-based eye trackers.

It should be noted that the physical properties in the list of central criteria vary between papers (see Table 1). For example, in the column ‘Minimum Speed’ some papers report the smallest peak speed of all detected microsaccades, whilst others report the smallest speed of a sample considered to be part of a microsaccade. Another option would be to report percentiles (e.g. 10 and 90) to give an idea of spread of the data whilst also protecting to some extent against outliers. Similarly, there are four different ways in which microsaccade distances may have been reported: (i) the Euclidean distance from the first to the last sample; (ii) the sum of the Euclidean distances between all consecutive samples (the integrated path length); (iii) the maximum Euclidean distance between any two samples in a microsaccade; or (iv) the difference between the points created by the most extreme horizontal and vertical positions, as suggested by Engbert and Mergenthaler (2006). If microsaccade paths are completely linear and recorded without noise, all these would be the same, but if microsaccade trajectories exhibit any form of curvature or overshoot there may be significant differences between the four possible metrics. 

Some physical properties of microsaccades appear to be consistently under-reported. Most prominently microsaccade duration is hardly discussed (7 out of 73 papers 17, 25, 62, 63, 64, 65, 66), although a lot of automated detection methods set minimum duration criteria (e.g. 63). Information about acceleration profiles of microsaccades is also reported only rarely (3 out of 73 papers 9, 48, 57). Some trackers might not provide sufficient temporal and spatial resolution for a meaningful calculation of acceleration; however, acceleration profiles might provide useful additional information for microsaccade detection. It would be advantageous to the field if more authors shared these details, so that microsaccades across experiments, trackers and external conditions could be more extensively quantified.

### Discussion

Based on this review the most promising physical characteristics of microsaccades to use in detection are (i) their velocity profiles, (ii) their binocularity, and (iii) their ballistic trajectories. All of these properties (to varying degrees of relative importance for the detection method) have been used successfully to detect microsaccades in the past. Microsaccade duration also stands out, as it is a parameter frequently used as an additional constraint on microsaccade detection, yet there is little evidence for which cut-off to use. In the future microsaccade detection has the potential to benefit from the accurate reporting of the physical parameters of microsaccades.

## Microsaccade Detection

The most commonly used microsaccade detection method of the last decade is the velocity-based EK method. As such, it is the most appropriate standard against which to compare other methods of microsaccade detection.

The EK method calculates a smoothed velocity trace in the horizontal and vertical direction separately and microsaccades are defined as samples in which the resultant radial velocity exceeds a threshold. Thresholds are based on a robust estimate of the standard deviation of the velocities (using medians) which can be modified using a multiplier (λ). On a polar plot of velocities, an elliptical boundary is used to accommodate differences between vertical and horizontal velocities. After thresholding, a minimum duration criterion is applied and only those microsaccades that occur in both eyes are considered. For different studies and tracker sampling frequencies some aspects of the method vary, such as the exact way of calculating the velocities, and the minimum microsaccade duration (5, 58). Many studies have reported adjustments to this method (e.g. 38, 67, 68). For clarity and consistency we decided to use the Microsaccade Toolbox for R, an online distributed version of the EK method (61). We have rewritten this code for Matlab, which we make available online as a supplementary file; along with example datasets.

Although widely used and accepted, the EK method is not without limitations. Since it relies primarily on velocity as an identifying feature of microsaccades, it is vulnerable to spurious increases in velocity resulting from various sources of measurement noise. To protect against inherent noise in the data, the EK method applies a fixed multiplier to the median-based velocity threshold for a particular dataset. The original 2003 paper further aimed to reduce misidentifications by requiring one sample overlap between microsaccades in both eyes. However, this binocularity criterion has been omitted when only monocular data was available (38, 63, 69, 70). Ultimately, using velocity as the main identifier of microsaccades may not be optimal and other avenues to detecting microsaccades should be considered.

Given the growing consensus that microsaccades occur simultaneously in the two eyes, it is interesting to evaluate the potential of a detection method based on binocular correlation between the eye movement traces. As microsaccades are also ballistic in nature, there must be some correlation between the speed profiles during microsaccades, even if the microsaccade might be larger or longer in one eye compared to the other, or if the microsaccade results in convergent eye motion. We would not expect this same rise in correlation between both eyes during drift and tremor, as they are largely uncorrelated. In this paper, the proposed BC method is compared to the EK method. The F1 score based on precision-recall (P-R) curves is used for assessing the performance of both detection methods compared to a ground truth based on manually coded microsaccades. The data used for evaluation came from two sources: a dataset specifically collected for this project and an existing published dataset from Nyström and colleagues (2017). We have made the data collected in our lab, as well as a basic example of the Matlab implementation of the BC method, available online as a supplementary file.

### Data Collection

#### Participants

Five observers with normal or corrected-to-normal vision were recruited for this research. They all gave informed consent and the experiment was conducted in accordance with the University of Oxford’s ethical review process. The first author was one of the participants in this experiment and is identified as P1.

#### Materials

Eye movements were recorded using an Eyelink 1000 eye tracker (SR Research) with a 25 mm lens. The tracker was brought to a distance from the eye of 55 cm for P1 and P2 and 35cm for P3-P5 to maximise the size of the pupil in the tracker’s field of view, for better microsaccade detection. The participants’ heads were kept stable using a chin and forehead rest. Participants viewed circular stimuli of different levels of Gaussian blur and matched summed intensities, which had a full-width at half-maximum of 19’ at a distance of 175 cm. Stimuli were generated using Matlab and displayed with 10-bit resolution on a Display++ driven via a ViSaGe graphics system (Cambridge Research Systems).

#### Procedure

Stimuli were presented for an initial duration of 2 s plus a randomly generated interval of up to 1 s. After this interval the target randomly moved up or down in steps of either 3’, 9’ or 27’ and the onset of movement was indicated to the participant using a beep. Observers were instructed to look at the target and follow it with their eyes. Between trials, 9 s of dynamic greyscale noise was displayed on the monitor to prevent afterimages. Participants completed 15 repeats of 9 trials of 5 s each, resulting in a total of 675 s of data per participant. Only the first phase before the target moved was considered in this analysis. This was done to remove the eye movements in response to the target movement which would contaminate the microsaccade trace. The instruction to follow the target was used to stop participants from focusing too heavily on keeping their eyes still. This resulted in a total of between 403 s and 409 s of data for each participant. An analysis of the data with regards to the blur of the targets and the following response to the movement of the targets will be published in a separate paper. 

The data collected by the above method will henceforth be referred to as the Oxford dataset. A published dataset by Nyström and colleagues (2017) (henceforth Lund dataset) was also included in the analyses to increase the variability of the data that was used to optimise the microsaccade detection methods. The subset of the Lund dataset used here contains manually labelled fixation data from 4 participants. The fixation stimulus was a white cross on a grey background viewed continuously for 200 s. Eye movements were recorded at 1000 Hz using the Eyelink 1000 Plus (SR Research).

### Data Analysis

#### Pre-processing

Before analysis, horizontal and vertical position data were first smoothed. Two methods of smoothing were compared: (i) the smoothing method supplied with the EK method published by Engbert & Mergenthaler (2006), which is a moving mean, and (ii) the Savitzky-Golay filter (3^rd^ order, frame length 21 ms) as described by Nyström and colleagues (2017), which is recommended because it preserves high frequency components of the signal well, without amplifying noise. Blink periods were removed, as well as 200 ms before and 300 ms after each blink, to avoid any unwanted artifacts from a partially occluded pupil.

Three different ways of detecting microsaccades were employed: manual coding, as well as the EK and BC methods.

#### Manual coding

**Figure 4.**
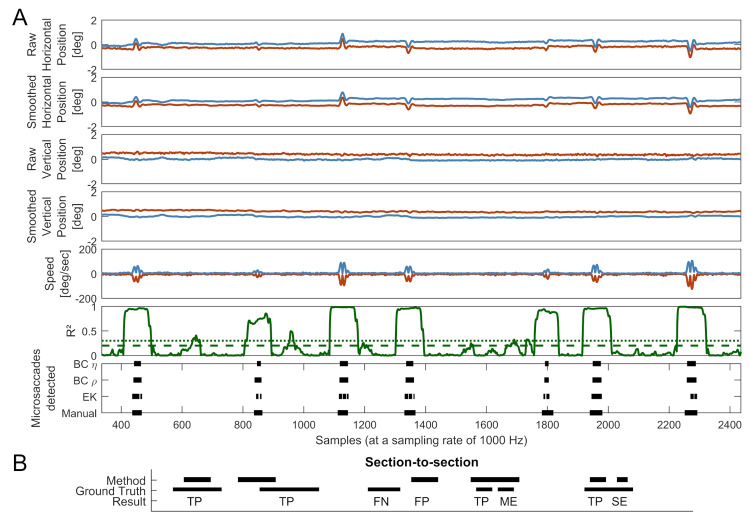
A: Comparison of microsaccade detection methods and their mapping onto smoothed gaze data, speed and correlation profiles for P4. The red lines indicate data from the left eye and the blue lines indicate data from the right. The green solid line represents the moving correlation of the speeds between both eyes with a window size of 65 ms. The corresponding finely dashed green line represents a η value of 7 and the coarsely dashed green line indicates a ρ value of .45. A total of just over 2000 samples or 2 seconds is shown. Note: Speed in the left eye is represented as negative to facilitate readability of the graph. B: Graphical explanation of how the ground truth and detection method were compared. TP: true positive; FN: false negative; FP: false positive; ME: merged event; SE: split event. Further explanation is given in the text.

For the Oxford data, the first author manually classified microsaccades. The procedure emulated that used in early experiments with analogue eye movement trace recording, where microsaccades were originally reported. Importantly, the manual coder was not given access to binocularity or velocity cues, so classification could remain as bias-free as possible. The full horizontal and vertical position traces up until the target movement were displayed for each trial, with data from each eye coded separately (e.g. Fig. 4 A, panels 1 and 3, where blue and red lines would be displayed separately). Microsaccades could then be selected by double clicking on the beginning and the end of each identified saccades. The height of each figure, corresponding to the vertical or horizontal eye position, was fixed to correspond to a minimum of 1° in size, ensuring that all displays were of the same maximum spatial resolution. For further analysis, the ground truth comprised only binocularly detected microsaccades (i.e. logical AND of the binary classifications for samples in the left- and right-eye traces). This decision was motivated by the consensus in the literature that microsaccades are binocular events. To protect against unfairly favouring the automated BC method in our analysis we additionally checked both automated methods (EK and BC) against a ground truth constructed from logical OR of the left and right eye coding.

For the Lund dataset manual microsaccade labels were taken from the downloaded dataset. The ground truth was constructed from binocular manual classifications by both coders (columns: ‘ms_type_c1’ and ‘ms_type_c3’) combined using a logical OR operation. This method of combining the two coders was chosen as it gave best performance of the EK method compared to using logical AND. Again, we produced a second ‘ground truth’ that included monocularly coded microsaccades, and used this to check whether good performance from the BC method was dependent on the way the ground truth was constructed. 

#### Automated EK

The EK detection method as published by Engbert & Mergenthaler (2006) was used for microsaccade detection and different values for lambda (3 to 9 in steps of .5) were considered.

#### Automated BC

To identify microsaccades using the BC method, moving correlations between the speeds in the left and right eye have to be calculated. The speeds in both eyes are the absolute velocities obtained from the first derivative as returned by the Savitzky-Golay filter, or the first derivative of the smoothed positions returned by the moving mean smoothing supplied with the EK method. To obtain radial velocities, as opposed to horizontal and vertical velocities separately, the square root of the sum of the squared horizontal and vertical velocities was taken. It is important to note here that the key variable is speed, not velocity, as it is not necessarily the case that the direction of the eye movement in both eyes is always well correlated during a microsaccade (42). In early piloting of this method we assessed if correlating the accelerations between both eyes could benefit the accuracy of this technique and found no improvement on microsaccade detection when including an acceleration criterion.

After extracting speed data, a threshold is applied to the coefficient of determination R² of speed between the two eyes. This results in a trace that has selected periods of high correlation, which depend in duration and prominence on the underlying microsaccade, as well as the size of the correlation window (ω) and prior smoothing. Too little smoothing leaves the data too noisy to analyse, while too much smoothing may remove all evidence of the smallest microsaccades or merge separate microsaccades, as well as artificially increasing correlation between both eye traces. Using larger ω values results in less prominent and wider periods of interest, as they correlate across more non-microsaccade samples. This, however, has to be balanced with the overall reduction in noise. As such, smoothing and the size of the correlation window ω interact in the BC method. This is akin to how data smoothing and the way of calculating the moving velocity in the EK method interact – some papers have proposed different velocity calculations to accommodate different tracking frequencies (e.g. 5). Both the EK method’s velocity calculation and the BC method’s moving correlation window essentially act as an additional level of smoothing.

Since the periods of high correlation extend beyond the microsaccade duration (see Figure 4A) by an amount that depends on the size of the correlation window ω, a set number of samples are removed from the beginning and end of the periods of high correlation. With a moving correlation window of width ω samples, the first correlation value to include at least one sample from a microsaccade will occur ω/2 samples before the start of the saccade. This increased correlation will extend to ω/2 samples after the end of the microsaccade. Therefore ω/2 samples are removed from each end of a period of above-threshold correlation resulting in the detected microsaccade.

A consequence of this is also that only microsaccades separated by more than ω samples can be detected as separate events. If they occur more closely in time than ω samples they will be reported as a single microsaccade. It is therefore important to choose a window width that is below the normal microsaccade interval time. The normal microsaccade interval, based on the microsaccade frequencies reported in the literature reviewed for this article ranges between 100 s (0.01 Hz) to 100 ms (10 Hz) with most articles citing values in the range of 2000 ms - 500 ms (0.5 - 2 Hz). This suggests that ω values below 100 ms are unlikely to produce a significant number of merged microsaccades. 

The method can be summarised in three steps:

I. Calculate the coefficient of determination (R^2^) for a moving correlation window between speed for left and right eyes.

II. Apply a threshold value to R^2^; high R^2^ values identify periods containing a microsaccade. 

III. Remove the first and last ω/2 samples from a period of above-threshold R^2^.

The BC detection method consequently has two main parameters that can influence the successful detection of microsaccades: the correlation window size ω and the threshold for speed R^2^ values. A threshold for the speed R^2^ values needs to be set, as this influences the number of identified microsaccades. There are two ways of choosing such a threshold value, both of which are explored. The first option is to select a fixed threshold value and apply this to all data. This method we refer to as BCρ, where the threshold value is specified by a parameter ρ for which threshold = ρ^2^. The second option is more similar to the EK method, in that a variable threshold value is chosen, based on a median multiplier, which we refer to as η, and consequently refer to this version of the BC method as BCη (where threshold = η * median of the speed R^2^ values). In the next section a systematic approach to optimizing these parameters is described.

### Evaluation Metrics

To compare the performance of the BC method with the established EK one, we used Precision-Recall (P-R) curves. P-R is an objective and established way to evaluate classification performance, but to our knowledge has not yet been used to quantify the performance of microsaccade detection methods, although it has been used in evaluating the similarity between manual coders (5,71). P-R curves plot precision (True Positives / (True Positives + False Positives)) against recall (True Positives / (True Positives + False Negatives)) and therefore give an indication of a method’s performance by relating how many correct identifications, misses and false positives occurred. As there is no perfect ground truth for which data samples represent a microsaccade, manually coded classifications were used, as they are the current gold standard to approximate ground truths. 

P-R performance can be summarised using the F1 score, which is given by:

**(1):**
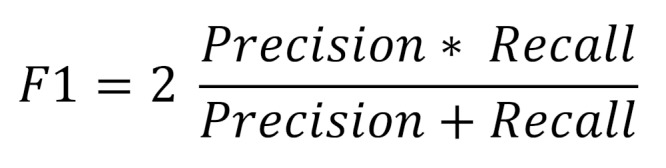


Precision and recall can either be specified on a sample-to-sample or section-to-section basis. When comparing methods on a sample-to-sample basis, each data point is assigned a binary value: 1 for being part of a microsaccade and 0 for not being part of a microsaccade, and true positives, false positives and false negatives are scored against the ground-truth. Sample-to-sample comparisons are sensitive to how accurately start and end points of saccades match between different detection methods. However, none of the three detection methods (including the manually coded ground-truth) is likely to be particularly accurate in identifying the edges of a microsaccade. Therefore, we chose to use section-to-section comparisons as a more robust method to indicate whether individual microsaccades were consistently identified.

Section-to-section comparisons assess if any microsaccade in the ground truth overlaps with any microsaccade detected by the detection method by at least one sample. Each data point is assigned a binary value and if there is any overlap between the ground-truth and method-detected microsaccade, this is counted as a true positive (TP). If there is a microsaccade in the ground truth that is not detected by the detection method, this is counted as a false negative (FN). Microsaccades detected by the detection method that are not present in the ground truth are classed as false positives (FP). There are two possible special cases: merged events (ME) and split events (SE). In a ME two or more microsaccades in the ground truth are represented as only one microsaccade by the detection method. This would be counted as one TP and one or more MEs. SEs occur when a single microsaccade in the ground truth is represented by two or more microsaccades in the detection method. This would be counted as a single TP and one or more SEs. For a graphical description of this, refer to Figure 4B.

Two different ways of considering MEs and SEs in the F1 scores were used: one in which MEs and SEs had no negative effect, and one in which MEs were treated as FNs and SEs as FPs. Not penalising the occurrence of SEs and MEs is reasonable, as the splitting and merging of microsaccades is a labile property of microsaccade detection. Researchers commonly manipulate it by merging microsaccades that occur within a given time of each other (72, 73), which changes the number of SEs depending on the interval used. Similarly, for the BC method, ω influences how many MEs are going to occur, as all saccades within ω of each other will be merged. However, as SEs and MEs are undesirable we repeated the calculations factoring in MEs as FNs and SEs as FPs, penalising their occurrence. This was done to check that the BC method was not overly impacted by the occurrence of MEs.

### Optimisation

As the BCρ method has two parameters that need to be set (ω and ρ), the interaction between different parameters was first freely explored using fixed values. Values of ω between 15 ms and 155 ms in 10 ms steps were considered. While 155 ms may have resulted in some merged saccades this space was explored as it may be the case that for an improvement in the detection of microsaccades some merging of microsaccades is acceptable. The values for ρ varied between .25 and .75 in .05 steps. This resulted in a total of 165 parameter combinations.

The parameter space for the BCη method was also explored, using the same ω values as for BCρ. The value of the median multiplier η was varied between 3 and 9 in .5 steps. For the EK method, the same values were used to vary the multiplier λ.

The two main aims for evaluating the new detection method were to find the best parameters to recommend for unknown data, as well as to give an estimate of how large the differences in detected microsaccades can be when transferring parameter estimates derived from one dataset to another. To find the best possible combinations of type of smoothing and detection parameters all combinations of parameters were considered and the combination of parameters that had the highest mean F1 score across participants (5 participants from the Oxford dataset and 4 from the Lund dataset) is the recommended combination. To give an estimate of how transferable optimisation is from one dataset to another, the method was trained on one of the two datasets (Oxford or Lund) and then tested on either itself or the other dataset. 

### Results

**Figure 5.**
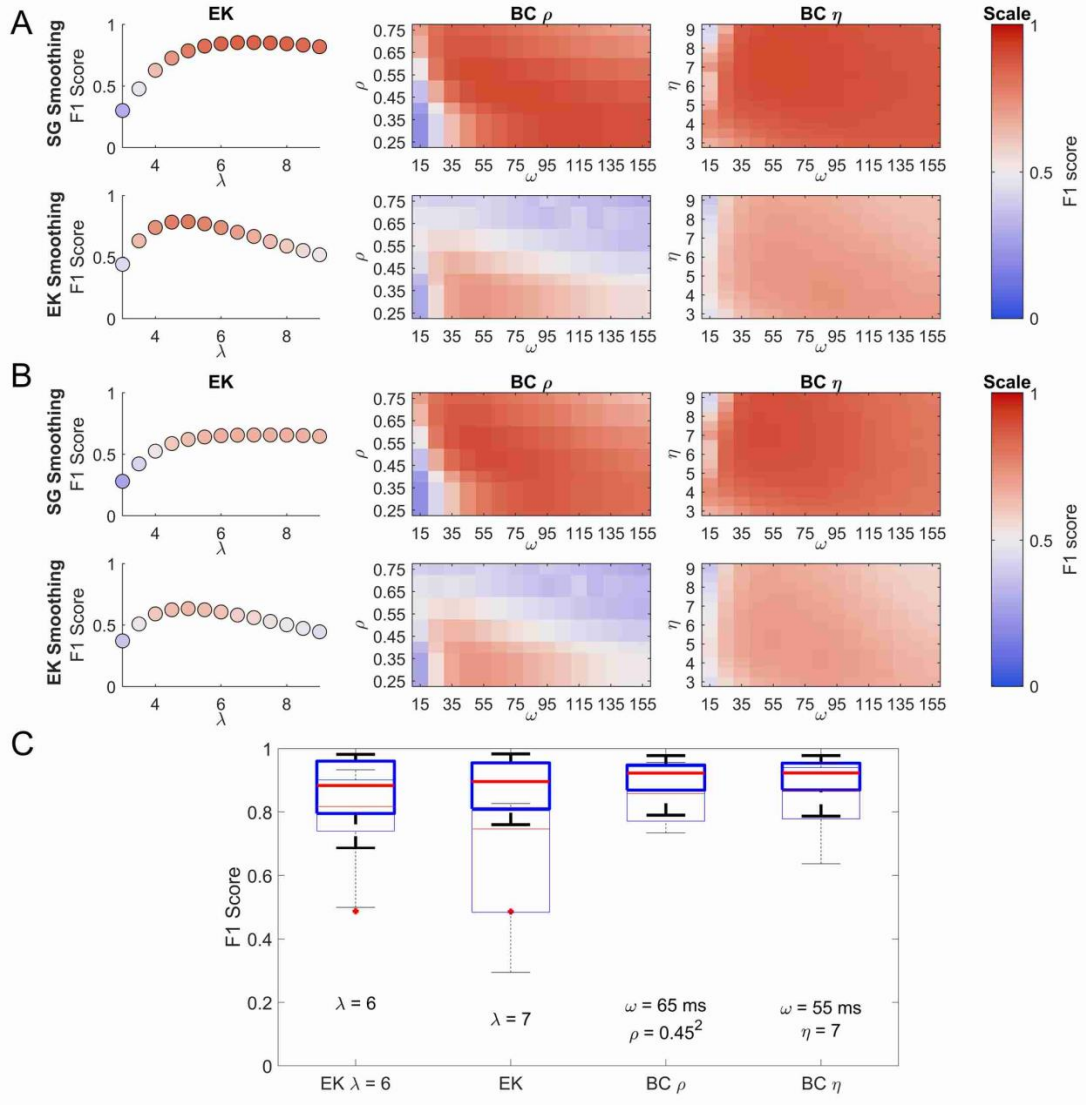
Comparison of the EK and the BC methods using the F1 score. Panels A and B show the F1 scores as a function of different parameter combinations for the three methods (EK:λ; BCη: ω and η; BCρ: ω and ρ), for this data smoothed using the Savitzky-Golay filter are shown above and data smoothed using the EK method are shown below. The colour map chosen is a perceptual colour map distributed by Kovesi (2015) under the name CET-D1. Panel A shows the F1 scores, ignoring MEs and SEs, while panel B shows the F1 scores when MEs are counted as FNs and SEs as FPs. The box plots in panel C show the spread of F1 scores for the 9 participants for Savitzky-Golay smoothing (5 from the Oxford dataset and 4 from the Lund dataset). For box plots the edges of the box represent the 25th and 75th percentiles, the line in the centre represents the median, while the errorbars show the most extreme values not considered outliers. Outliers are plotted as separate red plus symbols. The thinly-lined box plots represent the same parameter combinations but compared to a ground truth including both binocular and monocular microsaccades. Below the box plots the parameters used are given. They represent the optimal parameters across all participants, apart from the EK λ = 6, which is given for context, as λ = 6 is often a suggested starting value.

How the different parameters influence the F1 score performance of the EK, BCη, and BCρ methods can be seen in Figure 5 A & B. Savitzky-Golay smoothing results in appreciably higher F1 scores for the BC methods while for the EK method it appears to provide a larger plateau of performance. Figure 5 A shows F1 scores for which SEs and MEs were ignored. Since Savitzky-Golay smoothing gave universally better performance than EK smoothing, we focus on the Savitzky-Golay data in the following sections. The BC methods show relatively stable performance for ω > 25 ms. When considering MEs as FNs and SEs as FPs (Fig. 5 B) the F1 scores for the BC method remain relatively stable, with a slow drop of for larger values of ω, consistent with a small number of merged saccades. For the EK method a drop in performance due to an increase in the number of SEs can be seen. For the BC method the differences in the top F1 scores between the different calculations are small. Not penalising MEs and SEs results in a top score of 0.906 (BCρ) while penalising them results in a score of 0.893 (BCρ) for the same parameter combination.

The box plots of the participants’ F1 scores (ignoring SEs and MEs) for the parameters that give the highest mean F1 score across participants are shown in Figure 5 C. The corresponding parameter values for the EK method, the BCρ, and BCη methods are also given, as well as the standard recommended EK method with a λ of 6. Due to the small number of available participants with manually coded data no tests of statistical significance were conducted.

The subsidiary sets of box plots in Figure 5 C show performance evaluated against a ground truth that includes binocularly and monocularly coded saccades. The purpose of this analysis is to check that the relatively good performance of the BC method shown in the main analysis does not rest on the assumption of binocularity in the ground truth, and indeed that performance of the BC method is no more affected by this assumption than the EK method.

When considering the data smoothed using the Savitzky-Golay filter compared to the theoretically-motivated binocularly-coded ground truth, the best performing EK λ for 1000 Hz data was 7. For the BCρ method an ω of 65 ms and a velocity threshold, ρ of .45^2^ performed best, while for the BCη an ω of 65 ms and a median multiplier value, η of 7 were optimal. 

For the Oxford dataset, the resulting physical features of microsaccades determined using manually coded data, the EK method and the BC methods are given in Table 1 (shaded grey). Where minimum or maximum values are required, we extracted the highest or lowest values for each microsaccade and quote the average across microsaccades here. For microsaccade distance we used the distance of the diagonal spanning the smallest rectangle to contain all the data points, described in the corresponding point (iv) above.

Microsaccade main sequences were plotted for all Oxford participants (see Fig. 8). The main sequence plots for all detection methods appear to fall reasonably well on a line in the log-log plot, close to unity. The BC method includes some smaller and slower microsaccades, compared to the manual coder. These may be real microsaccades that are below the detection threshold of the manual coder but are successfully picked up by the BC method. If this were the case, these samples would lower the apparent precision value of the BC method, as they would be counted as false positives. Visual inspection of the data suggests that this is indeed the case. However, we cannot rule out the possibility that some of these may also be false detections.

Comparing the results of the P-R analysis for the manually identified binocular microsaccades to the results when using all manually identified microsaccades (binocular and monocular ones) might potentially provide information about the nature of classification errors. If all microsaccades are binocular events, spurious monocular classifications would arise where the signal is by chance above or below detection threshold in one eye or the other. The drop in the total F1 score when including monocularly coded microsaccades in the ground truth (Fig. 5 C) arises from a slight absolute increase in the correctly identified microsaccades (an increase in precision), but more of the monocular microsaccades are not detected by the automated methods (a decrease in recall). This suggests that some of the monocular microsaccades are true microsaccades, but that the majority are likely to be over-classifications by the coders. This may also explain why the automated detection methods have identified some smaller microsaccades when the binocular manual coding had not: if the trace was below the classification threshold of the coder in one eye only, but the relevant parameters are borne out in the data, then the automated methods may be able to detect the microsaccade.

**Figure 6.**
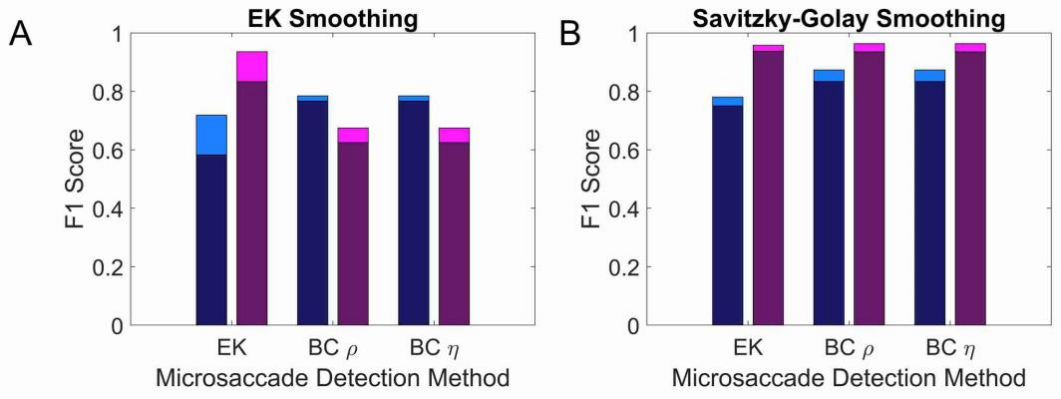
Transferability of the different detection methods between datasets. The deeply coloured section of each bar indicates performance when transferring the optimal parameters for one of the two datasets to the other, while the lightly coloured section of each bar indicates the added benefit from using the optimal parameters for each dataset. Results for the Oxford dataset are given in blue, while the results for the Lund dataset are given in purple. Panel A shows the transferability using data smoothed with a Savitzky-Golay filter and panel B shows data smoothed using the EK filter.

The transferability of the different detection methods from the Oxford to the Lund dataset and vice versa is shown in Figure 6. On visual inspection transferability appears to be better for data smoothed using a Savitzky-Golay filter compared to the EK smoothing.

**Figure 7.**
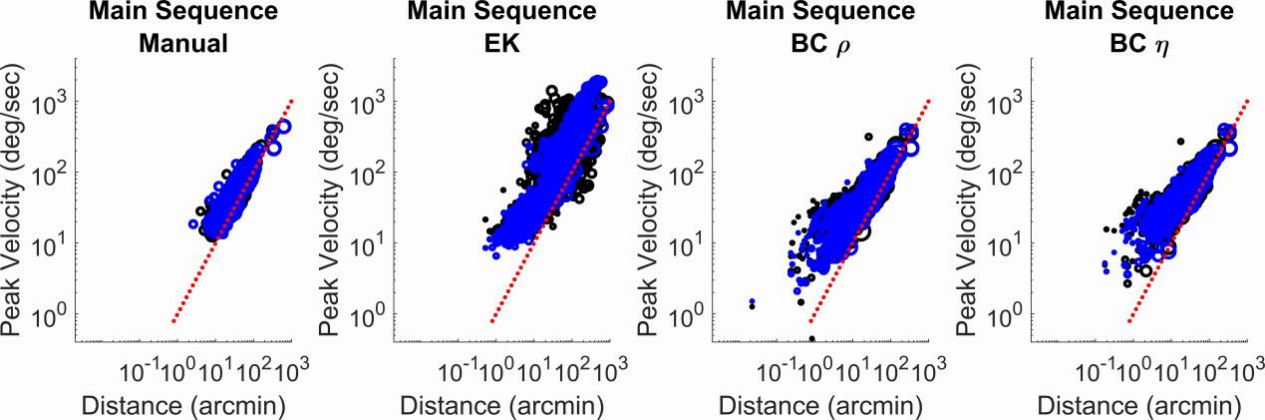
Microsaccade main sequences across all participants in the Oxford dataset for manually coded data, as well as microsaccades identified using the EK and the BC methods. The blue circles represent microsaccade detected in the right eye, while the black circles represent microsaccade in the left eye. The area of each circle is scaled in relation to the number of samples in that microsaccade. The red line represents the unit diagonal.

The analyses presented here suggest that, with Savitzky-Golay filtering, both the EK method and the BC method are likely to produce F1 scores above 0.8, and that the transfer to new datasets should be robust. 

### Discussion

The BC method presented here is a new way of classifying microsaccades. Because speeds in both eyes are more correlated during microsaccades than during drift and tremor these correlations can be used to successfully identify relevant periods in the data. Based on the data available for this study, the best parameters to use on unknown data are an ω of 65 ms and a fixed threshold ρ of .45^2^. Whilst there was no large difference between the BCρ and BCη methods, the box plots for BCρ were smaller, indicating a smaller interquartile range and therefore more consistency between participants, which is useful for microsaccade detection.

The analysis of classification performance as a function of parameter values (see Fig. 5 A & B) suggests that the BC method is fairly robust to the choice of parameters since, within a broad range of tested parameters, the F1 score remains high. If adjustments are necessary, it would be advisable to stay in the middle of the range of tested ω values. Too small an ω will result in some noise being misclassified as a microsaccade and too large an ω will produce lower correlation signals that may not be detected above the background values as well as merging microsaccades occurring close to each other. After choosing an ω value, good performance can be achieved by tuning the ρ value, as indicated by the shape of the surface in Figure 5 A & B.

The analyses presented in this paper also favour using a Savitzky-Golay filter for smoothing the data for both the EK and BC methods as F1 scores are typically higher. Also, with this smoothing the peak in F1 scores persists over a greater range of parameters and transferability between different datasets may be higher.

## General Discussion

Correctly identifying microsaccades is important in many areas of research, from trying to understand the visual system (28), to medical research (46) and studies on attention (74, 75). Over time our understanding of what makes a microsaccade has developed significantly, yet there have been few attempts to improve microsaccade detection based on our advances in knowledge. The EK method relies on the theoretical basis that microsaccades are outliers in velocity space and uses a simple binocularity criterion as a way of rejecting noise. The presented BC method relies on the temporally synchronous nature of microsaccades between the two eyes, and considers speed data, which is expected to be correlated between the two eyes during microsaccades. Other candidate characteristics for theory-driven methods of microsaccade detection are their characteristic ballistic motion, the main sequence relationship between their peak speed and distance travelled, as well as a combination of the physical parameters that limit them.

We evaluated the performance of the commonly used EK method, and a new method based on an increase in binocular correlation of speeds during microsaccades. In direct comparison, both microsaccade detection methods performed well on two separate sets of data, the Lund dataset (5), and the Oxford dataset. From the comparison to the default value of the EK method in Figure 5 it is obvious that a λ of 6 is a good starting value, which can be further improved with knowledge of the dataset. For the BCρ method, an ω of 65 ms and a fixed threshold ρ of .45^2^ are recommended.

If individual adjustments to ω are required, values above 25 ms appear acceptable. It is clearly visible in Figure 5 A & B that an ω of 15 ms leads to a considerable drop in performance compared to the other window sizes. The maximum size of ω should be below the interval in which two saccades may be expected, as any saccades occurring within ω ms will automatically be merged into one by the detection method.

The BC method focusses on a different feature of the microsaccade trace compared to the EK method and it may therefore be possible to choose which method to use depending on the experiment. For example, the BC method may be used when deliberately analysing only binocular microsaccades, while the EK method can be used to investigate potential monocular microsaccades or when binocular data are not available. 

Perhaps a technique similar to the unsupervised clustering method proposed by Otero-Millan and colleagues (2014) could be employed to optimise detection by combining the BC and EK methods. Preselecting candidate microsaccades using a liberal λ for the EK method and then choosing only those candidate microsaccades with the highest binocular correlation values may improve performance. One benefit offered by the binocular correlation method is that the height of the correlation peak may be used as an indicator of confidence in the correct identification of the microsaccade.

P-R is a useful tool for studying the effectiveness of microsaccade detection methods. No microsaccade detection method is without issue, including the manual detection used to generate a ground truth. However, using a weighted F1 score, it would be possible to select methods that are favourable because they are either biased to high recall or high precision. Having a high precision value may be useful in isolating only microsaccades from data, which may be particularly relevant for studies on covert attention, where drift periods are of no interest and contamination of data with them should be avoided. Alternatively, when research is interested in drift properties, and contamination with microsaccades should be avoided, biasing a method towards high recall may be beneficial.

One remaining challenge for microsaccade detection remains how to establish and test for the exact start and end points of microsaccades. While manual coders are likely to identify microsaccades well in the data, there is less reason to believe that their judgements of saccade start- and end-points are accurate. The same is true for microsaccade detection methods: falling below a velocity criterion or being less well-correlated are not intrinsic markers of the start and end point of microsaccades. For both of these measures the resultant points may be better represented for more prominent saccades, compared to small and slow saccades that are close to noise levels. A better understanding of microsaccades is required to detect these points well. Nevertheless, many papers report using a minimum duration criterion.

The BC method may also be beneficial in detecting larger saccades, which are always well-correlated between both eyes. The Oxford dataset includes some larger saccades, since we imposed no upper cutoff for microsaccade size, and there is good indication that the method works well for these examples. However, this has not been formally tested within the current project.

As the field of microsaccade research continues to grow and develop, reporting a variety of physical microsaccade parameters can contribute to the development of future detection techniques and increase the comparability of results between studies. It remains important for the progress of the field that we keep evaluating the research methods we use, from tracking setups to task design, participant instructions and detection mechanisms. One crucial side to this is that it is desirable for more researchers to make their eye tracking data available, including manual coding of microsaccades, where possible. Only with more diversity in the data available to train and test detection methods can we truly evaluate performance and make informed choices in the quest to optimise microsaccade detection.

## Ethics and Conflict of Interest

The authors declare that the contents of the article are in agreement with the ethics described in http://biblio.unibe.ch/portale/elibrary/BOP/jemr/ethics.html and that there is no conflict of interest regarding the publication of this paper. 

## Acknowledgements

This work was supported by funding from the Biotechnology and Biological Sciences Research Council (BBSRC) [grant number BB/M011224/1], the University of Oxford Wellcome Trust Institutional Strategic Support Fund (105605/Z/14/Z), and the John Fell Oxford University Press (OUP) Research Fund (103/786 and 151/139).

We wish to thank Martin Rolfs for support in accessing the reference list for his 2009 review, Marcus Nyström for help in accessing the Lund dataset.
